# Development of seismic vulnerability index methodology for reinforced concrete buildings based on nonlinear parametric analyses

**DOI:** 10.1016/j.mex.2019.01.006

**Published:** 2019-01-26

**Authors:** Moustafa Moffed Kassem, Fadzli Mohamed Nazri, Ehsan Noroozinejad Farsangi

**Affiliations:** a School of Civil Engineering, Universiti Sains Malaysia, Engineering Campus, 14300, Nibong Tebal, Penang, Malaysia; b Department of Earthquake Engineering, Faculty of Civil and Surveying Engineering, Graduate University of Advanced Technology, Kerman, Iran

**Keywords:** Seismic Vulnerability Index using NL analytical approach, Seismic vulnerability index, Vulnerability curve, Parametric modeling, Damage grade/state, Nonlinear analysis, Engineering demand parameter

## Abstract

This paper presents a simplified method in the seismic vulnerability assessment of reinforced concrete (RC) buildings based on proposed seismic vulnerability index (SVI) methodology. The employed procedure is derived with some modifications from the Italian GNDT and the European Macro-seismic approaches. Eight parameters were modeled in three distinct vulnerability classes to estimate the vulnerability indices of RC structures. The vulnerability classes were categorized based on the earthquake resistant design (ERD) defined as; (Low, Moderate, and High)-ERDs. Nonlinear time history analysis (NL-THA) and nonlinear static analysis (NL-SA) were carried out to define the weight of each parameter in order to calculate the seismic vulnerability index in a specific intensity (PGA) of an earthquake event. Knowing that it ranges from 0 to 1 from less vulnerable to most vulnerable with respect to the seismic intensity. In addition, the engineering demand parameter (EDP) used to determine the vulnerability index as the maximum top displacement of the structure. After determining the (SVI), The mean damage states were developed to evaluate the estimated physical damage of buildings in distinct seismic intensities.

• This simplified methodology helps to manage and implements strategies for the safety of the communities before earthquake takes place by investigating the vulnerability classes for each building type.
• Modeling the parameters that have an influence on the structural behavior without considering the past-damages observations through an analytical approach.
• Developing the seismic vulnerability index can reduce or limit the role of the rapid visual screening methods, which is based on expert opinion decisions, and depends on observations of damages caused by earthquakes, and can be a useful framework criterion in earthquake filed.

## Graphical abstract

**Figure:**
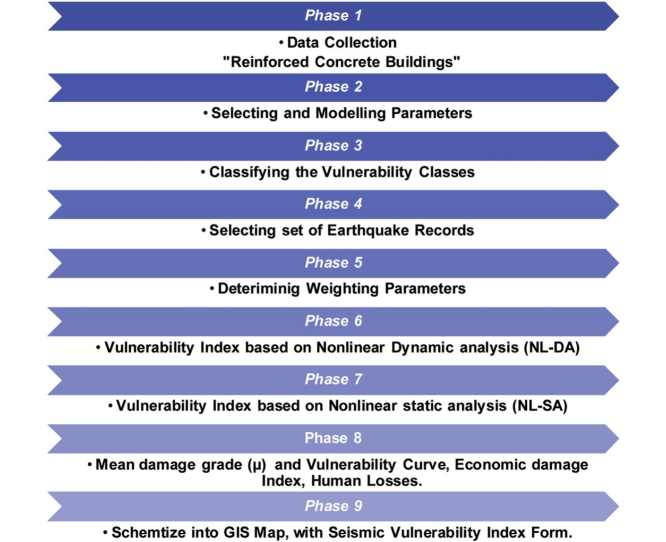

**Specifications Table**

**Table:** 

**Subject Area**	• Engineering
**More specific subject area:**	Civil Engineering, Structural and Earthquake Engineering
**Method name:**	Seismic Vulnerability Index using NL analytical approach
**Name and references of the original method**	• GNDT II approach, vulnerability Index (Iv) ***Reference:****Benedetti, Duilio, and Vicenzo Petrini. "Sulla vulnerabilitá sismica di edifici in muratura: Proposte di un metodo di valutazione." L’industria delle Construzioni 149 (1984): 66–74*. • RISK_UE project, European Macro seismic (EMS-98) approach, vulnerability Index (V). ***Reference:****Grünthal, Gottfried. European macroseismic scale 1998. European Seismological Commission (ESC), 1998.*

## Method details

**Fig. 1:**
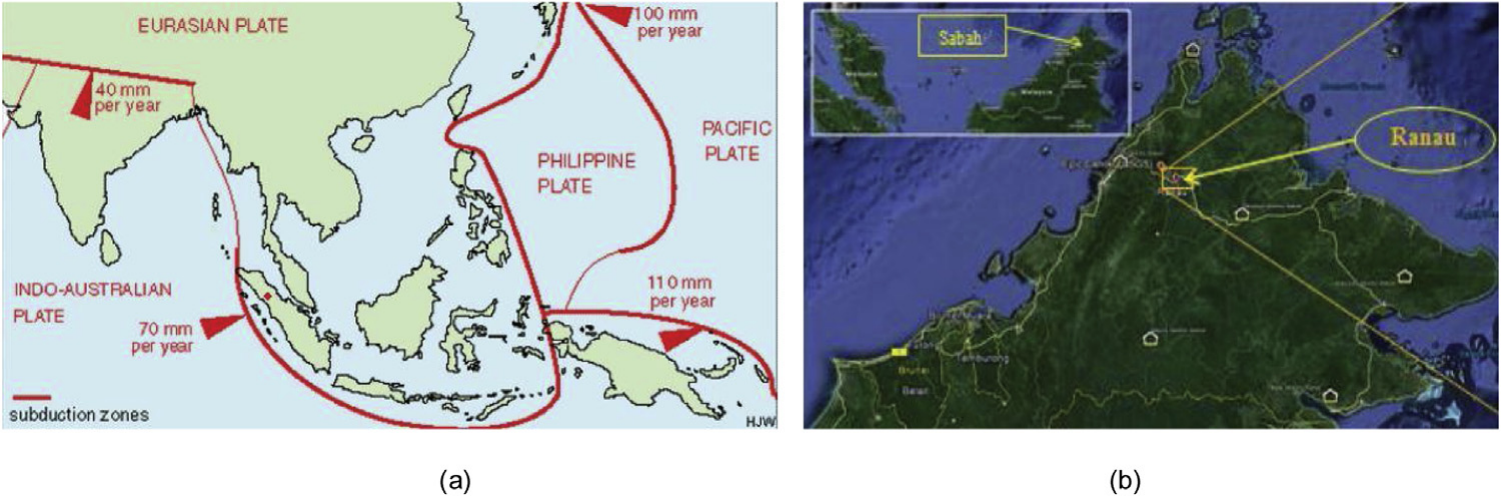
(a) Tectonic plates surrounding Malaysia, and (b) Location of Ranau earthquake hitting Sabah [2].

Nowadays, the issue of Malaysia’s safety from seismic tremors has been raised interest after an earthquake hit East Malaysia. In June 2015 as recorded, a moderate earthquake struck Ranau, Sabah with moment magnitude (Mw) of 6.0 in Richter scale, which has been the strongest earthquake affecting Malaysia since 1976. This has been due to the friction happened between the Philippines and Australia tectonic plates. What happened recently in East Malaysia is such a prove that Malaysia classified as having low to moderate seismicity and must consider the effect of earthquake loading in future edition of building design code [1]. Fig. 1(a) and (b) shows the tectonic plates surrounded Malaysia and the location of Ranau earthquake.

Earthquake phenomenan as a natural hazard is causing high levels of vulnerability and damages to the structures around the world. The reason behind is generally related to engineers or specialist who do not comply with the construction regulations to resist earthquakes or the seismic code guidelines for economic reasons. Nevertheless, there are several approaches that contribute in reducing the structural damages and have the capability to improve the seismic performance. One of these methods called the vulnerability index method which is used to express the damage level of an urban area on a large scale or even for one single building. Vulnerability index method is an empirical approach created in Italy by “The National Group of Defense from Earthquake” namely: GNDT, also created by the European Commission in seven European countries. This has been because of, no global program was developed in Europe, and due to the socio-economic and political impact of the seismic events that happened in Tukey, Athens, and Greece [3,4]. This method depends on a large amount of damaged data caused by pre-earthquakes that are needed to elaborate the most important parameters affecting and controlling the building structural vulnerability. Most of the recent studies were focusing on the seismic vulnerability of masonry structures, and few others were focused on steel structures [[5], [6], [7], [8]]. The methodology used in the previous studies is a combination of GNDT II and European Macro-seismic approaches. For example, in Portugal the GNDT II approach was used to assess the vulnerability of the masonry structures which overwhelmed in the Portuguese cities such as; Horta, Seixal, Faro and Coimbra, using the empirical vulnerability index, while in Spain the European Macro-seismic approach was applied [[9], [10], [11]].

The present methodology deals with the development of seismic vulnerability index (SVI) for a set of existing RC buildings in Malaysia using the Non-linear time history analysis (NL-THA) based on an array of earthquake ground motion records, and Non-linear static analysis (NL-SA) based on performance limit states and the plastic hinges formation affecting the structure, as well as to develop the vulnerability or fragility curves. In addition, to identify the economic damage index factor as the ratio of retrofitting cost to replacement cost as an estimation for the economic losses, as well estimating the rate of human casualties based on the probabilistic approaches. The following phases represent the structure of this research methodology that can be classified as follows:

Phase 1: Data collection of the RC-buildings in Malaysia to be analyzed.

Phase 2: Selecting and modeling the parameters affecting the seismic vulnerability.

Phase 3: Classifying the vulnerability into three classes as: Low ERD, Moderate ERD, and High ERD seismic resisting categories.

Phase 4: Selecting an appropriate set of ground motion records and apply the Nonlinear time history analysis using Finite Element software.

Phase 5: Determining the weighting parameters based on the top maximum displacement of the structure.

Phase 6: Calculate the Seismic Vulnerability Index (SVI) that ranges between 0 and 1 from less vulnerable to most vulnerable respectively, in different seismic intensities.

Phase 7: Calculate the Seismic Vulnerability Index (SVI) by applying the Nonlinear static analysis (NL-SA) based on plastic hinges formation in beams and columns.

Phase 8: Estimation of Mean damage state, Vulnerability Curves, Economic damage Index (EDI), and Human and homelessness losses.

Phase 9: Schematize the results in GIS form, and Develop Seismic Vulnerability Index form for surveying purposes.

## Strategy adopted for numerical modelling to define and calibrate the parameters

In order to develop a new approach that defines and calibrate the structural parameters influencing the vulnerability of RC-buildings, a number of parametric analyses were required to be carried out. Vulnerability scenarios are presented via FE model and the analysis results were assessed to define seismic vulnerability classes and weight of each parameter. Building typology and its traits are fundamental parameters to start the vulnerability evaluation, which represents a principle step to be considered. Most of the researchers deal with this idea where numerous parameters influence the physical vulnerability of the structure.

In this stage, the main concern is regarding the process adopted to develop the seismic vulnerability index of RC-buildings. The process followed the GNDT-II approach, with modifications through modeling some of the parameters, and by defining their weights or the coefficients into three distinct vulnerability classes; Low-ERD (L), Moderate-ERD (M), and High-ERD (H) from worst condition into best condition by utilizing NL platform. The Low class shows that the structure has not been designed incorporating seismic regulations and may have some deficiencies against the seismic loading. On the other hand, the High class shows that the building is properly designed according to seismic design code, and high performance to resist seismic loading, while the Moderate or the Intermediate class, where the parameter indicates moderate performance level against the seismic loading. Meanwhile, applying non-linear time history analysis (NL-THA) and non-linear static analysis (NLSA) via simulating a set of earthquake ground motion records can determine the parameters weight and their influence on the building seismic response, which in turn repeals the role of expert judgment through weighting parameters values in the GNDT-II. The parameters highlighting in the GNDT-II approach that is related to vulnerability index are illustrated in Table 1.

**Table 1:** Classes and relative weight of each parameter in the GNDT-II.

Number	Parameters	Classes Cvi			Vulnerability Index
		A	B	C	
1	Type and organization of the resisting system	0.00	−1.00	−2.00	$Iv^{*}=\sum_{i=1}^{i=11}Cvi$
2	Quality of resisting system	0.00	−0.25	−0.50	
3	Conventional strength	0.25	0.00	−0.25	
4	Building position and foundation	0.00	−0.25	−0.50	
5	Horizontal diaphragms	0.00	−0.25	−0.50	
6	Plan configuration	0.00	−0.25	−0.50	
7	In height configuration	0.00	−0.50	−1.50	
8	Connections and critical elements	0.00	−0.25	−0.50	
9	Low ductility elements	0.00	−0.25	−0.50	
10	Non-structural elements	0.00	−0.25	−0.50	
11	General maintenance conditions	0.00	−0.50	−1.00	

According to the aforementioned parameters, the proposed methodology focused on modeling eight parameters based on the three mentioned vulnerability classes. The eight parameters namely are; **(P1):** Beam-column joint connection, **(P2):** Boundary condition support, **(P3):** Horizontal Diaphragm system, **(P4):** Type of Soil, **(P5):** Ductility Level, **(P6, P7):** Horizontal and Vertical Irregularity in terms of mass ratios, and **(P8):** Concrete Strength. The hypothesis behind modeling the eight parameters is to develop the seismic vulnerability index (SVI) in different ground motion intensity (PGA), this procedure of modeling is explained below.

### (P1): Beam-column joint connection

The modeling of this parameter has been done according to the rigid offset length [12,13]. In class (L), the structures are modeled with no rigid offset length (β = 0) as flexible joint which is known by a simple shear connection or centerline model, by releasing moment and no rotation is allowed. In third vulnerability class (H), the structures have been modeled as fully rigid joint with rigid offset length (β = 1), which is known by fully restrained connection joint. While, in class (M), the structures have been modeled as semi-rigid joint with an offset length equal to 0.5, this type of joint known by partially restrained connection joint.

### (P2): Boundary condition support

Designing the structures against earthquake loading depends on the theory of dissipation in-elastic energy dissipation. Where the boundary condition is one the factors that affect the capacity of dissipated energy of the structure. It also can represent the ground conditions, in case of land sliding, subsidence and liquefaction effects [14,15]. This parameter is modeled according to the support of the structure to study the effect of the boundary conditions on the structure’s capacity. The modeling of the structures has been done in three vulnerability classes defined previously. In class (L), the structures are modeled with all hinged support or simply supported. In class (H), the structures are modeled with all fixed support or fully restrained, while in the second class (M), there are two cases which must be considered, the 1^st^ case is when the supports are externally fixed and internally hinged, and the 2nd case which the supports are externally hinged and internally fixed.

### (P3): Horizontal diaphragm system

A diaphragm is a horizontal planar system that serves to transmit lateral loadings to the vertical structural elements, as well as it supports gravity loads in case of out of plane bending, and the uplift forces due to vertical acceleration caused by earthquakes in near-fault regions. Under seismic loading, RC floors behave as a diaphragm to distribute the inertial forces that are generated by earthquake to the resisting frame and wall elements [16]. The relative stiffness of floor diaphragm with respect to the stiffness of the vertical resisting elements determines how the shear forces and torsional moments transfer to the lateral members and define the flexibility and rigidity of the floor. Where the in-plane stiffness of the floor as a diaphragm behavior plays an important role in transferring shear forces and torsional moments under seismic loading. The diaphragms are classified into three groups of relative flexibilities: Rigid, Flexible, and Semi-Rigid.

The horizontal diaphragm parameter has been modeled in three types and distributed into three vulnerability classes from worst (L) into the best (H) [17]. In the low class (L), the floor is modelled as a Flexible diaphragm where the out of plane stiffness can be ignored by modifying the out of plane stiffness modifier (M11, M22 and M12) which are responsible for bending and torsional forces, while in the high class (H), the floor is modelled as a Rigid diaphragm, with high in-plane stiffness and no deformation in the plan diaphragm, and in the moderate class (M), the floor act as semi-rigid diaphragm where half of the nodes are connected to the resisting elements.

### (P4): Type of soil

**Fig. 2:**
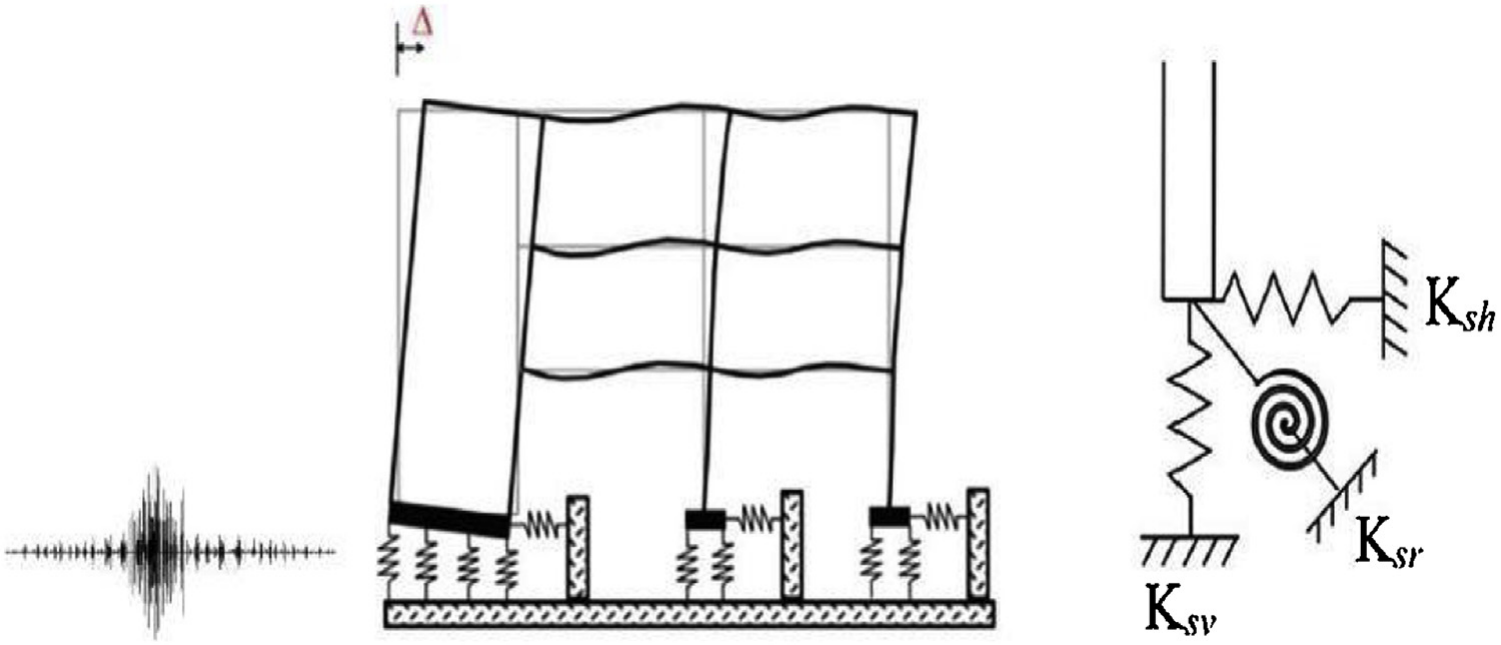
Schematic illustration of the SSI approach considered in this study [20].

Modeling the soil structure interaction (SSI) and its classification is applied as follow:

By Illustrating soil as spring model and determining its translational and rotational stiffness, the SSI is modeled as soil-spring supports estimated for foundations according to the soil type (C, D, and E) in NEHRP provisions [18]. The purpose of including this parameter is to estimate the influence of soil interaction type under the effect of seismic loading on RC buildings as shown in Fig. 2. To estimate the spring stiffness, the dimensions of the foundation should be defined, as well by determining the soil mechanical properties. The springs stiffness are calculated by defining the stiffness translation and rocking factors given in FEMA 356 in the following Equations [19]:

Translation along x-axis:

(1) $$Kx=\frac{GB}{2-v}\left[3.4{\left(\frac{L}{B}\right)}^{0.65}+1.2\right]$$

Translation along Y-axis:

(2) $$Ky=\frac{GB}{2-v}\left[3.4{\left(\frac{L}{B}\right)}^{0.65}+0.4\left(\frac{L}{B}\right)+0.8\right]$$

Translation along Z-axis:

(3) $$Kv=\frac{GB}{1-v}\left[1.55{\left(\frac{L}{B}\right)}^{0.75}+0.8\right]$$

Rocking about x-axis

(4) $$Krx=\frac{GB^{3}}{1-v}\left[0.4{\left(\frac{L}{B}\right)}^{0.75}+0.1\right]$$

Rocking about y-axis

(5) $$Kry=\frac{GB^{3}}{1-v}\left[0.47{\left(\frac{L}{B}\right)}^{2.4}+0.034\right]$$

Torsion about z-axis

(6) $$Krz=GB^{3}[0.53{\left(\frac{L}{B}\right)}^{2.45}+0.51]\;$$

Shear Modulus

(7) $$G=\rho s\;x\;Vs^{2}$$

Density of Soil

(8) $$\rho s=0.44\;x\;Vs^{0.25}$$

Allowable Bearing Capacity

(9) $$q_{allowable}=2.4\left(10^{-4}\right)\rho sVs$$

Where G: shear modulus, L: Foundation Length, B: Foundation Width, V_s_: Poisson’s Ratio.

### (P5): Ductility level

The ductility is defined as the capacity of a structure to undergo in-elastic deformation without rupture, otherwise, the structure is in the brittle stage. Most of the codes used different values of force reduction factor for the same structural system. This factor has different name in codes, behavior factor (q-factor) in Eurocode8, response modification factor in UBC97 code, response modification coefficient in ASCE7-16. For the current study, the ductility of RC building parameter is defined based on the response modification factor given in UBC1997 [21]. According to vulnerability classes, the low vulnerability class (L) is modeled as ordinary moment resisting frame (OMRF) building with response modification factor (R = 3.5), while in the high vulnerability class (H) it is modeled as special moment resisting frame (SMRF) building with response modification factor (R = 8.5). The moderate vulnerability class is defined as an intermediate moment resisting frame (IMRF) building having response modification factor (R = 5.5).

### (P6 and P7): Horizontal and vertical irregularity

In the current study, the mass irregularity is considered for the selected RC buildings. Modeling of this parameter has been done by considering the mass ratio, *m_r_*, in two different locations (Top floor and Bottom floor), which is well-defined as the ratio of the massive floor over the mass of an adjacent floor [[22], [23], [24]]. In addition, a regular model which signify as a controller (reference) model having a uniform distributed mass over the whole building is considered. The mass ratios are chosen to be variable values to represent the structural irregularity that must extend well beyond the limit of mass ratio (*m_r_*  = 1.5) that is adopted by UBC97 code. According to UBC97 provisions, the mass irregularity is considered to exist if the mass of any story exceeded 150% of the adjacent story. Thus, 6m_B_ denotes a building with mass ratio equal 6 on the bottom floor, as a nomenclature m_r_m_location_.

### (P8): Concrete strength

After an earthquake event, an assessment of the damaged building should be done. One of the critical reasons for structural damages can be related to concrete strength [25,26]. For this purpose, various concrete strength grades are designated to model this parameter. While considering concrete strength, the elastic modulus (E) is a necessary parameter to predict different strain values and to assess the deformations of the structures. As apart from the study, it is more conservative, to specify the concrete strength according to the regulations presented in seismic design guidelines. In the ACI code, the concrete strength is classified to be normal if the value would be 2500 psi (16 MPa) or lower, and to be adequate for earthquake resistance if it would be 5000 psi (35 MPa) or more. To this, the concrete strength parameter is modeled into three grades as; C16, C25, and C35 with respect to vulnerability class L, M and H respectively [27].

## Weighting the modelling parameters by nonlinear analysis

**Fig. 3:**
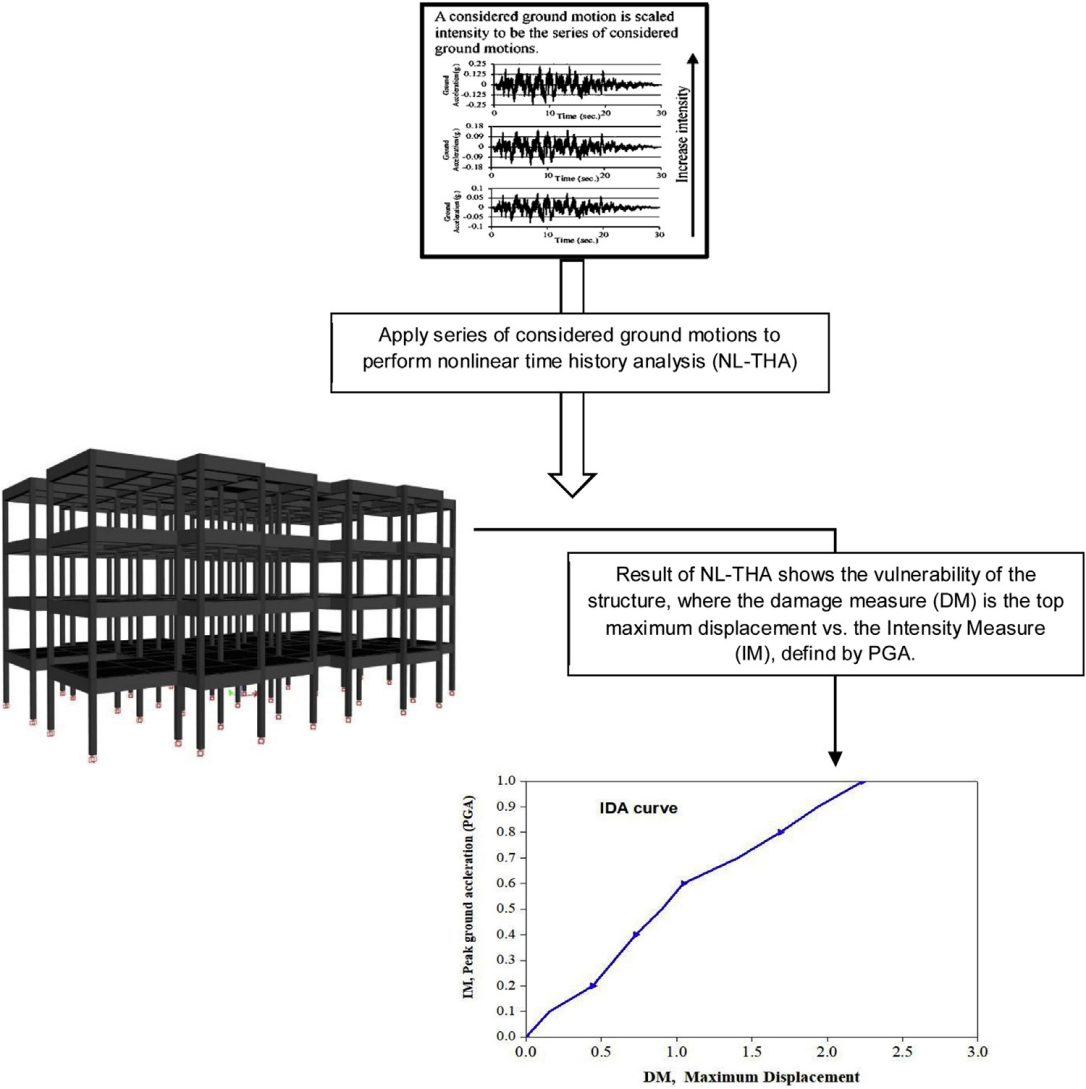
The concept of the incremental dynamic analysis (IDA) using ground motion records.

**Fig. 4:**
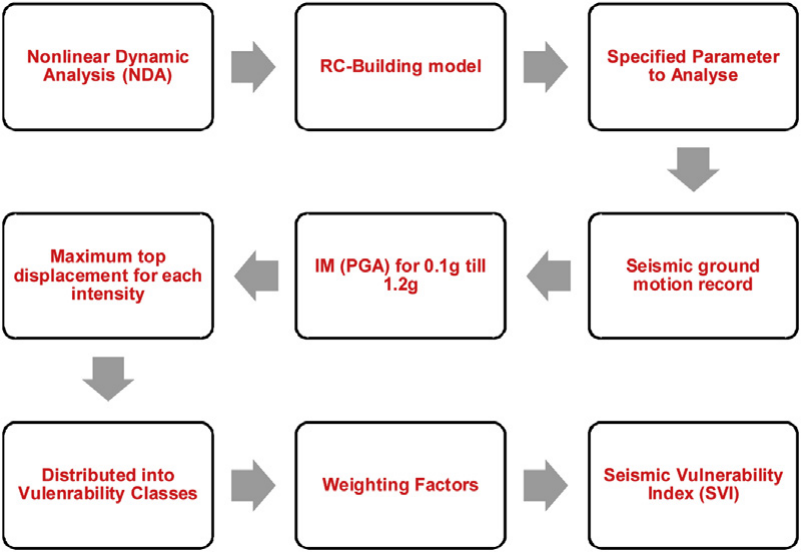
Flow-chart diagram to estimate the seismic vulnerability index (SVI).

To determine the weight of parameters, nonlinear time history analysis (NL-THA) and nonlinear static analysis (NL-SA) are utilized to extract the (IDA) and the (POA) curves, respectively. To define the weight of each parameter, the vulnerability of each structure is quantified by the maximum top displacement as tooled, that allows to estimate how the parameters influence the physical vulnerability of the buildings and their contribution on the response behavior during an earthquake. The damage state is expressed in the seismic vulnerability analysis via the evolution of the maximum displacement from linearity to nonlinearity till reaching the failure stage. This explains how the structure mechanism behave in three different vulnerability classes (Low, Moderate and High) from the worst parameter condition into the best condition, in terms of seismic loading. Fig. 3, Fig. 4, and Table 2 describe the procedure to quantify the seismic vulnerability index using NL-THA.

**Table 2:** Seismic vulnerability index estimation using (NLDA), in case of single building or group of buildings.

Weighting Factors per Parameter	Estimation of Seismic Vulnerability Index for a Single Building	Estimation of Seismic Vulnerability Index for a set of Buildings
Ki; is the displacement capacity ratio for each vulnerability class	$Ki=\frac{D_{\text{m}\text{a}\text{x}}}{\sum_{i=1}^{i=3}D_{\text{m}\text{a}\text{x}}}$	$Ki=\frac{D_{\text{m}\text{a}\text{x}}}{\sum_{i=1}^{i=3}D_{\text{m}\text{a}\text{x}}}$
Kj; is the average factor with respect to the number of analyzed buildings	None	$Kj=\frac{\sum_{i=1}^{i=n}Ki}{number\;of\;Buildings}$
K_L_; is the average factor with respect to the number of seismic records, (N = 7). *(From 3 to 7 records as a minimum requirement in seismic provisions to apply Nonlinear time history analysis).*	$K_{L}=\frac{\sum_{i=1}^{i=n}Ki}{Number\;of\;seismic\;records,\;\left(N\right)}$	$K_{L}=\frac{\sum_{i=1}^{i=n}Kj}{Number\;of\;seismic\;records,\;\left(N\right)}$
Kn, Final Weighting Parameter	The normalized factor is the value obtained by dividing the K_L_ for each parameter with the sum of K_L_ factors obtained from the vulnerability class L, to express the probability or percentage of seismic vulnerability for the RC-Buildings.	
	$K_{n}=\frac{K_{L\;}}{\sum K_{L\;},\;class\;L}$	
Seismic Vulnerability Index (SVI)	$SVI=\sum_{n=i}^{n=j}Kn$	

*D_max_ is the maximum top displacement for each vulnerability class (L, M, and H) at specific seismic intensity (PGA).

*∑ $D_{\text{m}\text{a}\text{x}}$ is the summation of the displacements in the three classes for a certain seismic intensity.

*N is the number of seismic records.

*n represents the number of parameters (n = 1–8) in this study.

## Seismic vulnerability index based on nonlinear time history analyses - IDA curves

Seismic Vulnerability Index based on Nonlinear Static Analysis (POA curves)

The Pushover analysis is a nonlinear static procedure in which the structural lateral loading increased incrementally according to the defined load pattern, under permanent vertical loads (dead load and live load). With increasing load, plastic hinges formation in the building frames and failure mechanism of the structure are found. The aim of the pushover analysis is to estimate the strength and deformation performance of the structural systems and comparing it capacities with performance levels according to ATC-40 and FEMA-237 criteria. This type of analysis would be obtained by plotting the base shear forces versus top displacement in a structure.

**Fig. 5:**
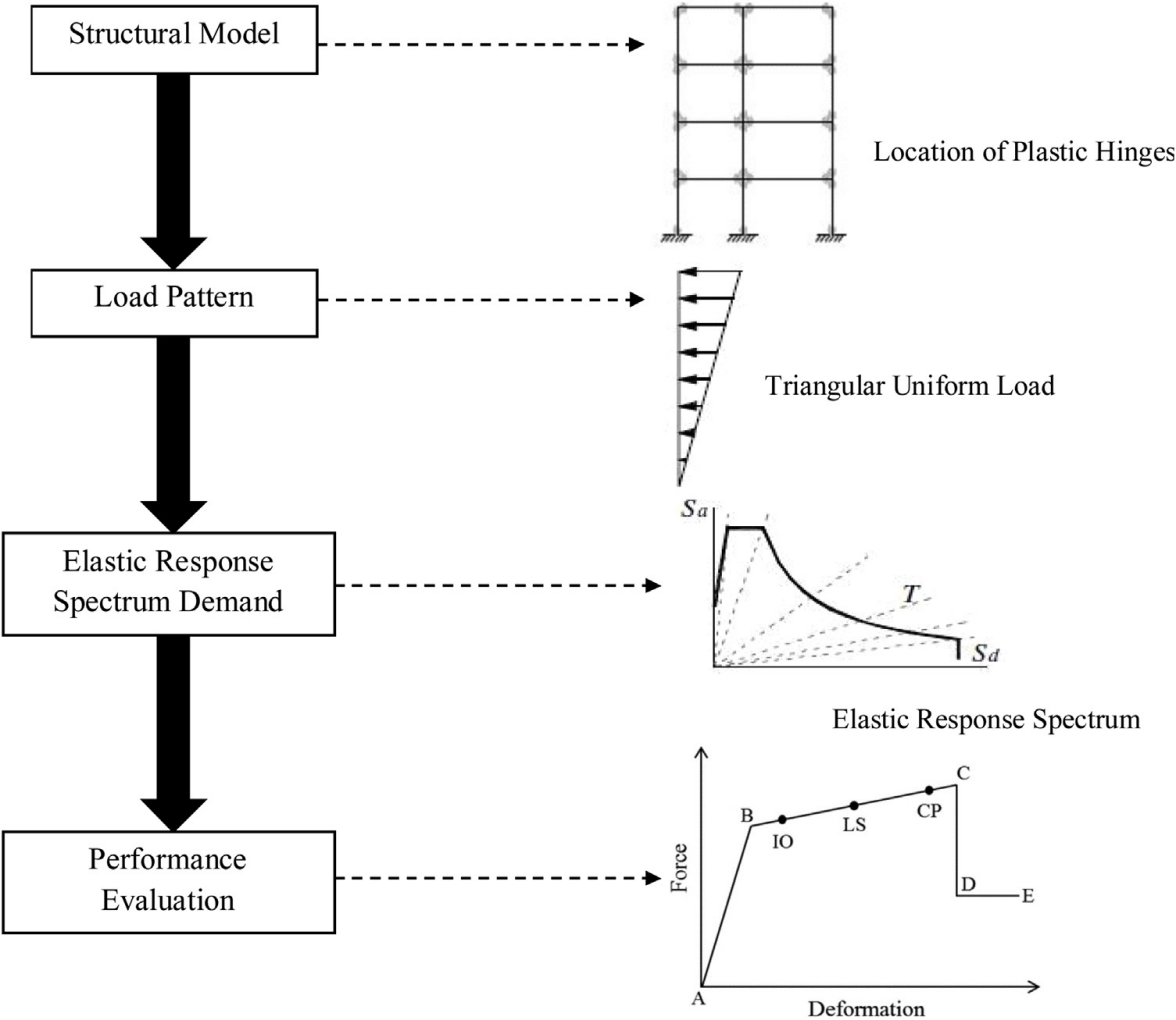
General flowchart for Non-linear static analysis procedure.

The nonlinear static analysis procedure contains 4 distinct phases as described below and illustrated in Fig. 5 [28]:

1 Develop the structural model and define the plastic hinges on the frame elements.
2 Define the lateral load pattern as an earthquake induce force.
3 Define the horizontal elastic response spectrum for Malaysia.
4 Estimate the performance level of the building, IO, LS, and CP, or with the five points labeled A, B, C, D, and E based on the acceptance criteria for the hinges.

## Seismic vulnerability index (SVI)

The damage occurred in the buildings during an earthquake can be measured using seismic vulnerability index (SVI). It is defined based on the weight factor of the frame elements (Beams and Columns), as well from the plastic hinges formation due to its performance levels. From the plastic plateau (B–C) in the load-deformation curve, can be subdivided into performance ranges namely, B-IO, IO-LS, LS-CP, CP-C, D–E, and > E.

After performing the pushover analysis, the number of hinges created in the frame elements of each performance level is needed to be calculated through the vulnerability index. As apart from the analysis, the weight factor (*x_i_*) is assigned to each performance level as shown in Table 3. The importance factor in the column element, is “1.5”, while in the beam element is equal to “1.0”. This is due to the fact that the global safety in the columns should be higher than beams. As a result, the seismic vulnerability index of a building structure is assessed by the expression given below:

(10) $${SVI}_{Building}=\frac{1.5\sum N_{i}^{c}x_{i}+1.0\sum N_{i}^{b}x_{i}}{\sum N_{i}^{c}+\sum N_{i}^{b}}$$

Where $N_{i}^{c}$ and $N_{i}^{b}$ represents the number of plastic hinges formed in columns and beams respectively, and I represents the performance level number, i = 1–6.

**Table 3:** Performance levels weighting factors [29].

Serial Number	Performance level (ith)	Weighting Factor (xi)
1	< B	0.000
2	B-IO	0.125
3	IO-LS	0.375
4	LS-CP	0.625
5	CP-C	0.875
6	C-D, D-E, >E	1.000

## Vulnerability classification of RC buildings

From the vulnerability index obtained by the nonlinear analyses, NLDA and NLSA, five vulnerability levels are proposed (Green 1, Green 2, Green 3, Orange 4, and Red 5) to evaluate the seismic performance of the buildings, this classification is illustrated in Table 4. Nevertheless, the vulnerability classifications were correlated with observed damage, that is described as; Negligible, Minor, Moderate, Severe/Partial Collapse, and Total Collapse as shown in Table 5.

**Table 4:** Reinforced concrete building vulnerability classification according to SVI [30].

Vulnerability Levels	Green		Orange		Red
	1	2	3	4	5
SVI	0.10–0.20	0.20–0.40	0.40–0.55	0.55–0.70	0.70–1.00
SVI, mean	0.150	0.300	0.475	0.625	0.850

**Table 5:** Vulnerability categories according to the observed damage [30].

Damage Categories	levels	Description
Negligible	Green 1	Negligible to light damage
Minor	Green 2	Light for structural elements, and moderate for non-structural elements
Moderate	Orange 3	Moderate for structural elements, and heavy for non-structural elements
Severe/Partial Collapse	Orange 4	Heavy for both the structural and non-structural elements
Total Collapse	Red 5	Total failure or collapse of the structure

## Mean damage state

Based on European Macro seismic approach EMS-98 scale, five damage grades are labeled as Slight, Moderate, Substantial to Heavy, Very Heavy and Destruction, denoted by D1–D5, respectively.

After defining the seismic vulnerability index (SVI) values for the RC buildings, it is essential to evaluate the mean damage grade related to each building. A mean vulnerability function is expressed to correlate seismic hazard with mean damage grade (0 < ${\text{μ}}_{D}$ < 5) of the RC buildings in a relation with the seismic vulnerability index (SVI) and corresponding to the seismic intensity (PGA) as shown in Eq. (11). The mean vulnerability function is adjusted into simple modifications utilizing the analytical approach instead of using post-earthquake damages observation and expert opinions [31]. For example, the seismic intensity used is associated for peak ground acceleration (PGA) instead of I_EMS-98_ scale, knowing that it is possible to establish a logarithmic relation between the seismic intensities to be correlated as shown in Eq. (12) and Table 6. In addition, expressing SVI instead of V to define the vulnerability index. With regards to that, Eq. (13) shows the correlation between the proposed methodology and the GNDT approach.

(11) $${\text{μ}}_{D}=2.839\;\times \left[1+\text{tanh}\left(\frac{PGA+10.79SVI-11.6}{Q}\right)\right]$$

(12) $$Ln\left(PGA\right)=a.I_{MCS}-b,\;where\;a=0.602,\;b=7.073$$

(13) V=SVI=-0.02+Iv.0.0104

Where PGA describe the seismic intensity of each ground motion record, SVI is the calculated seismic vulnerability index (SVI), *Iv* is the vulnerability index in GNDT approach and Q is the ductility factor of the construction typology ranging from 1 to 4, assumed to be 3 in this work.

**Table 6:** The correlation between the seismic intensities (PGA) and EMS-98 scale.

**PGA(g)**	**0.017**	**0.031**	**0.057**	**0.1**	**0.2–0.3**	**0.35–0.6**	**0.65–1.15**	**1.2**
**IEMS-98**	V	VI	VII	VIII	IX–X	X–XI	XI	XII

According to mean damage grade analytical expression ${\text{μ}}_{D}$, vulnerability curves function of mean damage grade vs. seismic intensity (PGA) for each building can be derived using the seismic vulnerability index (SVI) approach (Table 7).

**Table 7:** School building damage classification.

RC-Building damage classification	
SVI (NL-THA)	0.702
SVI (NL-SA)	0.693
Vulnerability Class	Red
Mean damage grade	Between D2 and D3
Iv (GNDT approach)	69

## Case study

**Fig. 6:**
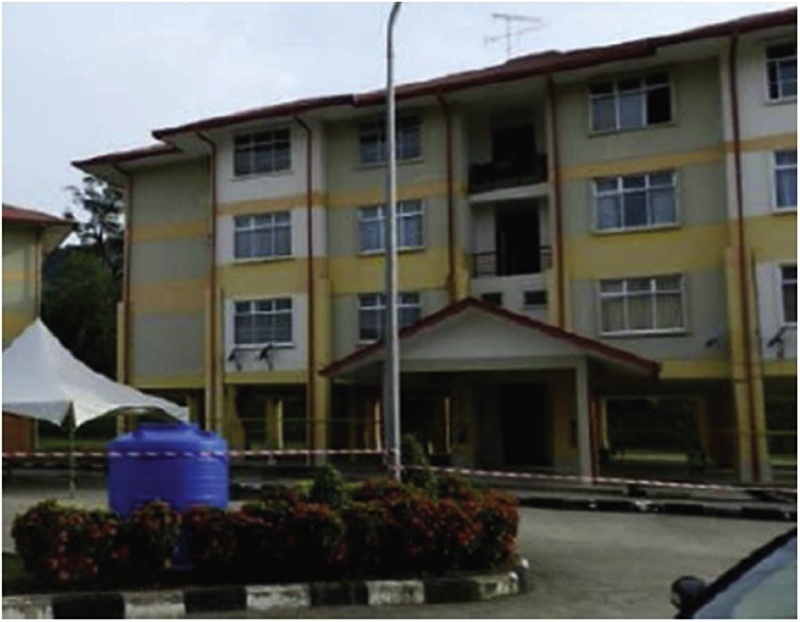
Selected reinforced concrete building.

**Fig. 7:**
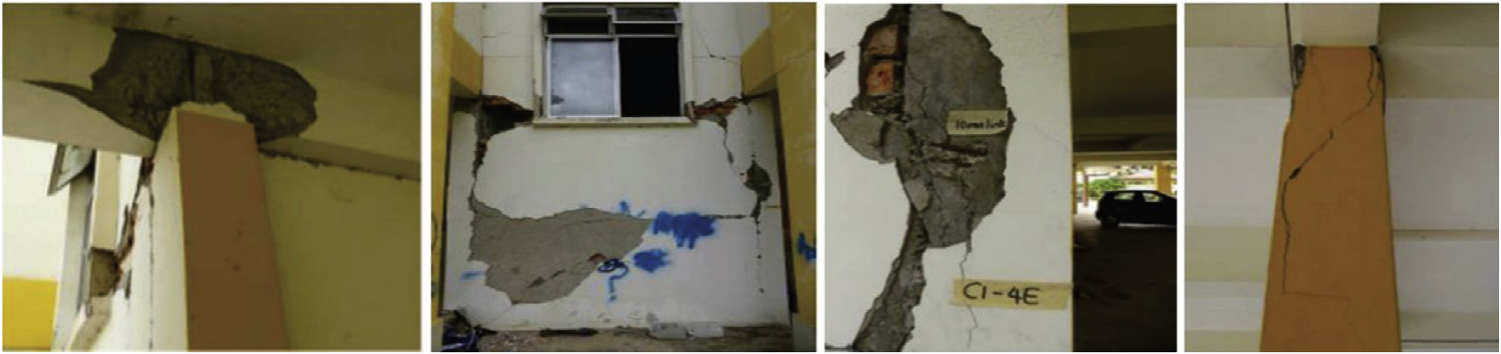
Field damage observation after Ranau seismic event [32].

The present case study is a 4-story reinforced concrete building classified as a “gravity load design” system and located in Sabah-Ranau, which is considered as a moderate seismic zone. The building is selected to verify the precision of the proposed methodology with the in-situ observations in terms of mean damage grade after determining its seismic vulnerability index (SVI). Fig. 6 shows the selected school building, and Fig. 7 shows the observed damage at the investigated school building due to Ranau earthquake.

After applying a set of ground motion records, the calculated SVI values due to nonlinear time history analysis (NL-THA) and nonlinear static analysis (NL-SA) are 0.702 and 0.693, respectively. Therefore, the building is set to be in the Red vulnerability class at a certain seismic intensity. Meanwhile, and based on the previous values of SVI, the mean damage grade of this building is determined to be very compatible with field observations where the damage distribution is between D2 and D3 damage state. The table below briefly illustrate the building damage classifications.
